# Eosinophils in inflammatory bowel disease pathogenesis: an ROS-centric view

**DOI:** 10.3389/falgy.2025.1608202

**Published:** 2025-08-08

**Authors:** Toshihiro Tomii, Gen Kano

**Affiliations:** ^1^ Department of Pediatrics, Kyoto Prefectural University of Medicine, Graduate School of Medical Science, Kyoto, Japan; ^2^ Department of Pediatrics, Japanese Red Cross Kyoto Daini Hospital, Kyoto, Japan

**Keywords:** eosinophil, gastrointestinal homeostasis, inflammatory bowel disease, reactive oxygen species, Siglec-8

## Abstract

Eosinophils (Eos), long recognized for their roles in allergy and helminth defense, are now emerging as key players in gastrointestinal immune regulation. In inflammatory bowel disease (IBD), eosinophils are frequently elevated in both blood and intestinal tissues, yet their functional significance has been underexplored. This review reexamines the role of eosinophils in IBD pathogenesis, integrating recent insights into mucosal immunity and tissue homeostasis. We outline the shift in perspective from viewing eosinophils solely as inflammatory effectors to recognizing their dual roles in inflammation and repair. Clinical and experimental findings reveal correlations between eosinophil abundance, activation markers, granule protein release, and disease activity in IBD. Central to our model is the regulatory function of eosinophil-derived reactive oxygen species (ROS), particularly hydrogen peroxide, in maintaining intestinal barrier integrity. Dysregulation of ROS—due to dysbiosis or genetic variants—may impair healing and exacerbate inflammation. We further highlight Siglec-8, an inhibitory receptor on eosinophils that induces apoptosis in response to Neu5Ac-containing sialic acids. This pathway may be disrupted by Neu5Gc, a non-human sialic acid abundant in red meat, potentially linking Western diets to impaired eosinophil regulation. These findings suggest new therapeutic directions targeting Siglec-8 and ROS balance to modulate eosinophil activity and restore intestinal immune homeostasis in IBD. These insights may also help bridge traditionally distinct disease paradigms by highlighting a potential common pathogenic mechanism of epithelial barrier dysfunction and dysregulated eosinophil activation shared between allergic diseases (e.g., asthma, eosinophilic esophagitis) and IBD.

## 1 Introduction

Eosinophils (Eos) are multifunctional leukocytes traditionally known for their roles in parasitic infections and allergic responses. However, accumulating evidence implicates eosinophils as active players in the pathogenesis of inflammatory bowel disease (IBD), which includes ulcerative colitis (UC) and Crohn's disease (CD) (1). Under homeostatic conditions, eosinophils normally reside in the gastrointestinal (GI) tract (especially the lamina propria of the intestine)—in fact, among healthy tissues, the GI tract harbors the highest number of eosinophils (with the exception of the esophagus, which normally lacks eosinophils). This basal presence suggests eosinophils contribute to maintaining mucosal homeostasis, beyond their classical anti-helminth or allergy-promoting functions. Indeed, a paradigm shift is underway: eosinophils are now recognized not only as pro-inflammatory cells in IBD but also as modulators of tissue repair and immune regulation in the gut.

Recent research highlights two emerging concepts in eosinophil biology relevant to IBD. First is a reactive oxygen species (ROS)-centered model of intestinal immune regulation. Traditionally, ROS generated during inflammation were viewed solely as harmful byproducts causing tissue damage. New evidence challenges this view, showing that controlled ROS production (e.g., by NADPH oxidases in epithelial cells and phagocytes) is essential for intestinal homeostasis (2). Eosinophils, with their potent ROS-generating enzymes (such as NADPH oxidase and eosinophil peroxidase), may act as key regulators of mucosal ROS levels, thereby influencing epithelial integrity, microbial balance, and healing processes. Second is the discovery of Siglec-8, a sialic acid-binding immunoglobulin-like receptor expressed on human eosinophils (and mast cells), which transduces inhibitory signals. Engagement of Siglec-8 on eosinophils leads to their apoptosis, functioning as an “off switch” to curtail eosinophil-induced tissue damage (3, 4). Intriguingly, this regulatory pathway involves recognition of sialic acids (notably N-acetylneuraminic acid, Neu5Ac) that may be released from injured tissue, implying a feedback mechanism where eosinophils limit their own aggression in response to host damage. Understanding how Siglec-8 is triggered and modulated (for instance, by different sialic acid forms present in diet and tissue) is a novel frontier in IBD research.

This review provides a comprehensive examination of eosinophils in IBD pathogenesis, integrating classical knowledge with recent advances in ROS-mediated immune regulation and Siglec-8 biology. We begin by discussing the changing perspective on eosinophil function in the intestine, then summarize clinical and experimental evidence linking eosinophils to IBD. We then propose a model of intestinal homeostasis centered on ROS, with eosinophil-derived ROS balancing mucosal injury and repair. Next, we explore the role of Siglec-8 and sialic acid interactions in regulating eosinophil activity and ROS in the gut, including how dietary influences (Neu5Ac vs. Neu5Gc sialic acids) might affect this pathway. Finally, we outline future directions and therapeutic implications, such as Siglec-8 agonism and dietary or glycan-based interventions, to modulate eosinophils in IBD.

Our aim is to provide an up-to-date, integrative view that not only underscores eosinophils as important contributors to IBD pathology but also as potential key regulators of intestinal immune homeostasis, offering insights for innovative therapies in allergic and inflammatory GI diseases.

## 2 Changing perspective on the role of eosinophils in the intestine

Eosinophils have long been viewed through the lens of helminth defense and allergic disease. In the gut, their classical role was attributed to protection against parasitic infections—a notion supported by the Th2-dominated responses (IL-4, IL-5, IL-13) and elevated eosinophil counts observed in helminthiasis (5–8). Indeed, eosinophils can directly kill parasites or aid in worm expulsion by degranulating toxic cationic proteins, generating ROS, and forming extracellular traps (9–11). However, in the absence of parasites, the presence of abundant eosinophils in intestinal tissues raised questions about their function. For decades, eosinophils in IBD were often considered “bystander” cells or merely a component of an inappropriately activated Th2 response. This simplistic view has been revised as research uncovers eosinophils' nuanced roles in both inflammation and homeostasis.

One shift in perspective is the recognition that intestinal eosinophils are normal residents with homeostatic roles. Even in healthy individuals, eosinophils populate the intestinal lamina propria from duodenum to colon (12), implying a role in baseline mucosal immune surveillance and tissue maintenance. They interact with the microbiota and other immune cells, contributing to the steady-state balance. For instance, as functions independent of parasite infections, eosinophils have been shown to support IgA production and maintain gut plasma cells via secretion of survival factors (like APRIL and IL-6) in mice, thereby reinforcing the mucosal barrier against microbes (13, 14); although there are some contrary evidences (15–19). Eosinophils can also produce trophic factors (e.g., epidermal growth factor, TGF-β) that influence epithelial growth and repair (20), as suggested by studies of eosinophil-deficient animals which exhibit impaired tissue homeostasis in the gut (21). Such findings underscore that eosinophils are integrated into the intestinal immune system as modulators of both immunity and tissue integrity. Another evolving concept is eosinophils' role as immune regulators during infection and inflammation beyond helminths. For example, in certain bacterial infections, eosinophils are now recognized to modulate host responses. Eosinophils can phagocytose bacteria (albeit less efficiently than neutrophils) and form DNA traps that ensnare microbes (22, 23). In Helicobacter pylori infection of the stomach, eosinophils become activated and may contribute to a Th2-biased microenvironment that promotes chronic infection persistence by dampening strong pro-inflammatory (Th1) responses (24). Eosinophils are also found to participate in granuloma formation in mycobacterial infections, where they help wall off bacteria but can also drive fibrosis through type 2 cytokines (25). These examples illustrate that eosinophils can have dual functions—protective or pathogenic—depending on context: they can aid in pathogen containment but also exacerbate tissue damage or fibrosis if over-activated or if pathogens resist clearance. Such insights have broadened our view of eosinophils from mere effector killers to complex immunomodulatory cells that can shape the overall immune response.

The intestinal environment further influences eosinophil function. Commensal microbiota appear to regulate eosinophil development and recruitment to tissues. Germ-free mice or those with altered microbiota show changes in gut eosinophil numbers, suggesting microbiota-derived signals (such as microbial metabolites) help set basal eosinophil levels (26). Conversely, eosinophils might influence microbial composition; eosinophil-deficient mice have reported shifts in gut microbiota (19), though causality is still under investigation. This bidirectional interaction hints that eosinophils might contribute to maintaining a healthy symbiosis with gut flora, whereas dysbiosis could alter eosinophil behavior.

Recent findings have highlighted the role of tuft cells in the intestinal epithelium as key initiators of type 2 immunity involving eosinophils. Tuft cells are chemosensory cells detect microbial or parasitic signals and secrete interleukin-25 (IL-25), which activates group 2 innate lymphoid cells (ILC2s) (27). In turn, ILC2s produce IL-5 and IL-13, promoting eosinophil recruitment and activation, thereby supporting mucosal defense and tissue remodeling (28). This IL-25–ILC2–eosinophil axis, initially characterized in the context of helminth infection, also extends to bacterial infections. In *Clostridioides difficile* infection (CDI) colitis models, IL-25 expression is suppressed, and IL-25 supplementation restores eosinophil numbers and reduces mucosal injury—despite no changes in bacterial burden or toxin levels (29, 30). Furthermore, clinical studies have shown that patients with peripheral eosinophilia at the time of CDI diagnosis experience lower mortality and fewer complications, suggesting eosinophils serve a protective function during infection (31). Collectively, these data position IL-25-induced eosinophil responses as a key element in preserving epithelial barrier function and resolving tissue inflammation during intestinal infections.

Furthermore, eosinophils can secrete anti-inflammatory and pro-resolving mediators—for example, they are a source of specialized pro-resolving mediators (SPMs) like maresins and protectins (32), as well as TGF-β, all of which can facilitate repair of damaged mucosa (33). Notably, eosinophil lifespan and survival can be significantly altered by the local cytokine environment; cytokines such as IL-5, GM-CSF, and IL-33 prolong eosinophil survival by delaying apoptosis and enhancing their functional capacity (34, 35). Conversely, resolution-phase mediators, including eosinophil-derived protectins and maresins, help dampen inflammation and support resolution processes, potentially creating a microenvironment less supportive of prolonged eosinophil survival (36, 37). Thus, context-dependent regulation of eosinophil longevity critically influences whether these cells play a predominantly pathogenic or reparative role. A balanced perspective is therefore emerging: eosinophils are a double-edged sword in the gut, capable of driving inflammation and tissue injury when dysregulated, but also essential for restoration of homeostasis and mucosal healing when properly regulated.

In summary, the role of eosinophils in the intestine has expanded from a narrow focus on parasite defense to a broader appreciation of their part in immune modulation, host-microbiome interactions, and tissue homeostasis.

## 3 Eosinophil involvement in inflammatory bowel disease: recent clinical evidence

Building on the concept of eosinophils' dual roles in gut inflammation and repair, we turn to their specific impact in inflammatory bowel disease (IBD). In IBD management, achieving mucosal healing has emerged as a paramount therapeutic goal, strongly associated with sustained remission and improved long-term outcomes (38). Standard therapy such as mesalazine (5-aminosalicylic acid, 5-ASA) is widely used to dampen mucosal inflammation and oxidative stress (39); however, simply scavenging reactive oxygen species and suppressing inflammation with 5-ASA may not be sufficient to fully restore the integrity of the gut lining or prevent future relapses. This recognition has spurred interest in other immune mediators that contribute to mucosal healing. Among these, eosinophils stand out as important yet underappreciated players in IBD, often overshadowed by the emphasis on neutrophils and lymphocytes in intestinal inflammation (1). However, eosinophils have long been reported to be involved in the clinical course of inflammatory bowel disease and have recently received renewed attention as they continue to accumulate. This section will present clinical evidence from diverse aspects showing an association between eosinophils and inflammatory bowel disease.

### 3.1 Peripheral blood eosinophilia in IBD

The correlation between eosinophilia in peripheral blood and disease has been recognized since very early times, for example, Machella and Hollan reported that steroid-refractory ulcerative colitis shows eosinophilia at the initial diagnosis (40). Recent large cohort studies confirm that peripheral blood eosinophilia (PBE) (often defined as >0.5 × 10^9^/L) is more prevalent in IBD than in healthy controls. For example, an Israeli nationwide study of ∼28,000 IBD patients found PBE in 13% of cases vs. 5% of controls (*p* < 0.001), with UC (16%) and pediatric IBD (24%) showing higher rates than Crohn's disease (CD) or adult-onset IBD (41). Importantly, in this study PBE tends to correlate with more aggressive disease: over multi-year follow-up, those with PBE experienced earlier hospitalization and surgery (including accelerated time to colectomy in UC) compared to non-eosinophilic patients. On multivariate analysis, baseline eosinophilia was an independent predictor of severe disease course (hazard ratio ∼1.5) as defined by steroid dependence, need for multiple biologics, or surgery. It also predicted earlier use of corticosteroids and biologic therapy. Another study analyzed registry data from the UC patients in United States and reported that peripheral eosinophilia had higher clinical activity indices, more frequent C-reactive protein elevation, and greater healthcare utilization (42). These findings support using blood eosinophil count as a prognostic marker for IBD severity. Nevertheless, not all studies agree. A smaller cohort of newly diagnosed UC patients saw no significant association between baseline blood eosinophil counts and histologic severity or long-term outcomes (43). Thus, while peripheral eosinophilia often signals higher inflammatory burden and worse prognosis in IBD, its predictive value may vary, and normal eosinophil counts do not rule out severe disease.

### 3.2 Tissue eosinophilia in the gut

Eosinophils in intestinal tissues are a well-recognized feature of IBD pathology, with counts typically increasing in active inflammation (44). Recent studies reinforce that mucosal eosinophil infiltration correlates with disease activity in many patients. For example, a 2023 prospective study in Indonesia reported a moderate positive correlation (*r* = 0.396, *p* = 0.005) between colonic eosinophil density on biopsy and UC clinical severity (by Truelove-Witts score) (45). Histologically, active IBD lesions often show numerous eosinophils clustering in the lamina propria and even within crypt abscesses or ulcerated areas, where they release eosinophil cationic protein and other granules. In both UC and CD, peak tissue eosinophil counts can reach very high levels during active flares. This tissue eosinophilia has been linked to outcomes: one pediatric IBD cohort found that higher baseline gut eosinophils associated with earlier need for corticosteroid therapy (46). Similarly, in adults, dense colonic eosinophils during active disease were associated with poor response to initial mesalamine/steroid therapy and with non-response to vedolizumab at 6 months (47). UC patients in endoscopic remission who still had residual mucosal eosinophilia tended to experience higher relapse rates (48, 49). These data suggest tissue eosinophils might drive ongoing smoldering inflammation.

On the other hand, there is some conflicting evidence on whether eosinophils signify worse disease or, alternatively, an attempt at mucosal repair. A few reports noted that UC patients (children or adults) whose biopsies showed low eosinophil counts (eosinopenic inflammation) actually had more severe, refractory disease requiring treatment escalation (50, 51).

This paradox aligns with the idea that eosinophils could also play roles in wound healing or immune regulation. Work by Lampinen et al. demonstrated that during UC remission, eosinophils remain present and even in an “activated” state (see below), whereas neutrophils largely disappear (52). Thus, context matters: mucosal eosinophilia generally reflects active IBD inflammation and often correlates with disease severity or risk of relapse, but an absence of eosinophils might in certain contexts signify a different, possibly more destructive inflammatory phenotype.

### 3.3 Activation markers on eosinophils

Activated eosinophils upregulate distinctive surface markers which can be assessed in blood or tissue samples. Key markers of eosinophil activation include the adhesion molecule CD44 and the β2-integrin CD11b (part of Mac-1), while CD9 (a tetraspanin) is high on resting eosinophils and diminishes with activation, so a CD44^high^/CD9^low^ profile is characteristic of activated eosinophils (53, 54). Using this characterization, a flow-cytometric study in IBD has shown that eosinophils from patients (especially during active disease) exhibit significantly higher CD44 expression compared to healthy controls (55). The percentage of CD44^high^ eosinophils was elevated in active UC and CD, and interestingly was highest in UC patients in remission. In contrast, Crohn's disease patients in remission showed a decline in eosinophil activation (CD44 levels) to near-control levels.

Not only surface markers but also certain histologically stained intracellular components can be regarded as activation markers, since activated eosinophils undergo degranulation and release granule proteins detectable by specific immunostaining. Indeed, immunohistochemical studies have demonstrated increased expression of eosinophil cationic protein (ECP) within eosinophils infiltrating the lamina propria in active ulcerative colitis (UC). These activated eosinophils significantly correlated with tissue inflammation severity and were associated with enhanced production of reactive oxygen species, indicated by nitroblue tetrazolium (NBT) reducing activity in severely inflamed mucosa (56). Another eosinophil-specific enzyme, eosinophil peroxidase (EPO), also showed markedly elevated levels during active UC, directly implicating eosinophils in mucosal damage mediated through oxidative stress (57).

Interestingly, a recent study revealed that PD-L1^+^ eosinophils—identified as active eosinophils with a T cell regulatory function via single-cell transcriptomics in a mouse model of colitis (more on this later)—exhibited significant abundance in PD-L1 samples from patients with ulcerative colitis (UC) and Crohn's disease compared to healthy controls (58). These findings collectively underscore the involvement of activated eosinophils primarily in inflammatory exacerbation and oxidative tissue damage in active IBD, but also in promoting tissue healing.

### 3.4 Eosinophil-derived cell free granule proteins in tissue and bodily fluids

Activated eosinophils release an array of toxic granule proteins—notably eosinophil cationic protein (ECP), eosinophil-derived neurotoxin (EDN), major basic protein (MBP), and eosinophil peroxidase (EPX/EPO)—which can be detected in tissues, blood, stool, or other fluids in a cell-free form. Studies in the last decade have explored these eosinophil products as biomarkers of IBD activity.

• **Fecal Eosinophil Cationic Protein (fECP)**: Levels of ECP in stool are significantly elevated in patients with active IBD compared to healthy individuals. While fecal calprotectin (neutrophil-derived) is the gold-standard fecal marker, fecal ECP has shown complementary value. A 2019 study of young adults with IBD by Abedin et al. found fECP was high not only in active UC/CD but even in some clinically inactive patients, indicating ongoing eosinophil activity (59). Notably, among patients in clinical remission with low calprotectin, an elevated fECP identified those who would soon require treatment escalation or even surgery; over 4 years, the probability of needing therapy intensification rose from 22% (if fECP <200 µg/kg) to 82% (if fECP >600 µg/kg) in this study. This suggests fECP can serve as a prognostic marker: high eosinophil protein release in an ostensibly quiescent patient signals smoldering disease and higher relapse risk. While fECP is less accurate than calprotectin for differentiating active vs. inactive disease (AUC ∼0.77 vs. 0.88) in Abedin et al. study, there is another report claiming that fECP has greater sensitivity and specificity than fecal calprotectin or myeloperoxidase (neutrophil origin) (60).
• **Serum ECP**: Eosinophil cationic protein can also be measured in blood. In active IBD, serum ECP levels are significantly higher than in healthy controls or IBD patients in remission. This was shown in a 2017 study that also noted serum ECP correlates with disease activity indices (61). However, serum ECP is a fairly nonspecific marker of eosinophil load and is influenced by atopic conditions. Its main clinical utility may lie in identifying a subset of IBD patients with a prominent eosinophilic component. Elevated serum ECP usually normalizes with successful treatment and remission.
• **Fecal Eosinophil-Derived Neurotoxin (EDN)**: EDN in stool has emerged as a particularly promising predictor of relapse in UC. A Swedish prospective study (2019) followed UC patients longitudinally and showed that fecal EDN levels rose significantly up to 3 months before a clinical relapse (62). A doubling of EDN concentration corresponded to a 31% higher risk of relapse (and similarly a doubling of ECP gave ∼27% higher risk). At the time of flare, EDN was markedly elevated compared to remission, but importantly EDN was also elevated during the predictive window prior to symptoms. This temporal relationship positions EDN as a noninvasive biomarker for impending flare in UC. (Interestingly, in Crohn's disease the same study found an inverse trend for EDN: patients who went on to relapse had lower EDN than those who remained in remission, underscoring again the differing inflammatory profiles of UC vs. CD.) Pediatric data likewise suggest that EDN can serve as a relapse indicator in eosinophil-rich intestinal inflammation (63).
• **Eosinophil Peroxidase (EPX)**: EPX is another toxic granule enzyme that generates reactive oxidants. Local EPX release in the gut has been linked to tissue injury in IBD. Studies have found colonic mucosal biopsies in active CD and rectal perfusates in active UC contain high EPX levels compared to inactive disease. One report noted EPX is significantly upregulated in intestinal tissues at initial IBD diagnosis and then tends to decrease during the disease course (64). This could reflect mucosal eosinophil degranulation being most intense early, or alternatively, mucosal remodeling reducing eosinophil recruitment over time. In a mouse colitis model, eosinophil peroxidase was shown to contribute to tissue damage (EPO-deficient mice had less colitis), reinforcing that EPX can actively drive inflammation (65). In other animal model, dogs with untreated inflammatory bowel disease had significantly higher numbers of degranulated eosinophils in the lower region of the lamina propria and both degranulated and intact eosinophils in the upper region compared to control and treated dogs, which is detectable by EPX staining of the small intestine.
• **Major Basic Protein (MBP)**: MBP is highly cytotoxic to parasites and host cells; it has been observed histologically alongside ECP/EPX, and increased MBP deposition was noted in the small intestines of patients with eosinophilic gastroenteritis (correlating with disease severity). We have fewer recent clinical studies quantifying MBP in IBD patients. One study measured eosinophil granule proteins in gut lavage fluid and although increased EDN and EPX was retrieved from active IBD patients, MBP was not found both in patients and controls (66). Another recent study revealed that abundance of MBP in esophageal biopsy can distinguish eosinophilic esophagitis (EoE) from IBD-associated eosinophilia in children (67). These findings suggest that the mode of MBP release is one of the key determinant of clinical phenotypes of 2 overlapping diseases in IBD and EoE.

In summary, the presence of eosinophil granule proteins in stool or tissue offers objective evidence of eosinophil activation in IBD. Fecal ECP and EDN, in particular, have shown value for monitoring disease activity and forewarning relapse. High baseline levels of these proteins in patients in remission portend a higher likelihood of flare, whereas declining levels correlate with mucosal healing. Such biomarkers are gaining traction as noninvasive tools to complement endoscopy in IBD management.

To better elucidate the role of each eosinophil granule protein, recent research proposes that variations observed in eosinophil-derived biomarkers across clinical contexts may stem from their distinct secretion mechanisms and functional specificities. Eosinophils employ multiple modes of granule protein release, including classical exocytosis, piecemeal degranulation, cytolysis, and eosinophil extracellular trap cell death (EETosis), allowing selective deployment of specific granule proteins in response to localized inflammatory signals and environmental conditions (68, 69). Among these mechanisms, piecemeal degranulation preferentially releases eosinophil cationic protein (ECP) and eosinophil-derived neurotoxin (EDN), potentially rendering these proteins particularly effective biomarkers of ongoing inflammation. Notably, EDN possesses a comparatively lower electric charge than ECP, enhancing its recoverability from tissue surfaces and bodily fluids (70). Additionally, EDN exhibits more efficient release from activated eosinophils than ECP, further substantiating its superior clinical utility in inflammatory assessments (71). Moreover, EDN demonstrates exceptional stability across diverse storage conditions, reinforcing its reliability as a clinical biomarker (72). Galectin-10, uniquely released during EETosis, has emerged as a distinct biomarker indicative of this specialized form of eosinophil cell death, often evidenced by the presence of Charcot–Leyden crystals (CLCs) in affected tissues (73). Although galectin-10 and EETosis have demonstrated diagnostic potential in asthma, eosinophilic esophagitis, and other eosinophilic disorders (74), there remains a significant gap regarding their role in inflammatory bowel disease (IBD). Current literature on galectin-10 in colitis is sparse, with only a solitary recent report identifying galectin-10 and associated CLCs in stool samples from patients suffering from clozapine-related eosinophilic colitis (75). Consequently, the prospective value of galectin-10 and EETosis as biomarkers or mechanistic components in IBD pathogenesis remains an open area for future research and cannot currently be conclusively determined from existing data.

### 3.5 Eosinophil-Related cytokines and chemokines

The recruitment and activation of eosinophils in IBD are orchestrated by a network of Type 2 cytokines and chemokines. Notably, interleukin-5 (IL-5) is the primary eosinophil growth and survival factor, and eotaxin-1 (CCL11) is a key chemokine attracting eosinophils to gut tissue (76–78). Recent investigations have measured these mediators to understand their clinical significance in IBD:

• **IL-5 and IL-13**: UC has long been associated with a skewing toward a Th2 immune response (in contrast to the Th1 bias of Crohn's). Lamina propria cells from UC patients produce higher IL-5 (and also IL-13) than those from CD [Lampinen et al. (55)]. IL-5 levels in colonic tissue or blood correlate with eosinophil counts. IL-13, produced by Th2 cells and type 2 innate lymphoid cells, can act on epithelial cells and is thought to contribute to UC pathogenesis (e.g., epithelial barrier dysfunction). Both IL-5 and IL-13 promote tissue eosinophilia: stimulating eosinophils with these cytokines *in vitro* causes upregulation of activation markers and degranulation. Clinically, the persistence of IL-5/IL-13 in mucosa may explain why eosinophils remain activated even during UC remission. A recent development is the use of periostin as a surrogate marker of IL-13/Th2 activity in UC. Periostin is a matricellular protein induced by IL-13; a 2025 study found serum periostin levels in UC correlate strongly with tissue eosinophil infiltration (79). High-periostin patients (i.e., “Type 2–dominant” UC) had significantly better clinical remission rates on corticosteroids, suggesting that identifying an IL-13/eosinophil-high endotype could inform treatment stratification. This underscores that Th2-eosinophilic inflammation in UC might be especially steroid-responsive, and periostin or IL-5/IL-13 levels could serve as biomarkers to predict therapy response.
• **Eotaxins (CCL11, CCL24, CCL26)**: Eotaxin-1 (CCL11) and its family members are critical chemokines that selectively attract eosinophils *via* the CCR3 receptor. Biopsy studies have shown eotaxin-1 is elevated in IBD mucosa with active eosinophil infiltration (Carlson et al. (64)). In both UC and CD tissue samples, areas with high eosinophil density often exhibit locally increased expression of eotaxin and related chemokines like CCL5 (RANTES). A study using Luminex technology aimed to assess cytokine and chemokine profiles in ulcerative colitis (UC) patients found that eotaxin-1was significantly increased in both serum and tissue of patients with active UC and correlated with disease severity (80). Serum eotaxin-1 was also examined in the context of therapy response: in the 2022 vedolizumab-response study, higher baseline eotaxin-1 levels were observed in patients who went on to respond to vedolizumab, compared to non-responders (49). Responders had a median eotaxin-1 of 0.33 ng/ml vs. 0.20 ng/ml in primary non-responder. Clinically, there is interest in blocking eotaxin to reduce eosinophilic inflammation. Bertilimumab, a monoclonal antibody against eotaxin-1, has been tested in a Phase 2 trial in ulcerative colitis (81).
• **Other Eosinophil-Related Mediators**: IL-33 (an epithelial-derived alarmin) can also promote type 2 responses and eosinophil recruitment; Recent studies have shown IL-33 to be upregulated in epithelial cells and myofibroblasts in UC (82, 83). RANTES (CCL5) and MCP-3/4 (CCL7/CCL13) are chemokines found elevated in IBD tissues alongside eosinophils (84). They likely contribute to recruiting not only eosinophils but other leukocytes. The aforementioned Luminex study (Coburn et al. (80)) also showed significantly increased G-CSF in UC patients' serum. While G-CSF has been shown to increase the adhesion of eosinophils, evidence of its other effects on eosinophil is scarce (85).

So far, most of these cytokines and chemokines have been studied more in mechanistic contexts than as clinical biomarkers. But a broad takeaway is that IBD patients with a “Type 2 high” immune profile—high IL-5/IL-13, abundant eotaxins, and eosinophils—form a distinct subgroup. This subgroup (more common in UC) may have different clinical behavior and treatment responses. For instance, as noted, they might respond better to steroids or therapies targeting lymphocyte trafficking, whereas “Type 1” dominant patients (more TNF, IL-12/23, neutrophils, but few eosinophils) might preferentially respond to other biologics.

Bringing these findings together: eosinophil-related parameters have shown significant correlations with IBD severity, activity, and outcomes. High blood eosinophil counts at diagnosis flag patients at risk for a more severe disease course (with earlier hospitalization, biologic needs, or surgery). Likewise, abundant tissue eosinophils in active disease often indicate intense inflammation that may be harder to treat. Persisting eosinophils in endoscopically inactive tissue forewarn mucosal immunologic activity that can herald relapse. Correspondingly, eosinophil degranulation markers in stool (like EDN and ECP) have predictive value for upcoming flares in UC. On the other hand, a paucity of eosinophils in colon biopsies has, in some studies, signified a more immunodeficient type of inflammation associated with poor healing. This duality suggests eosinophils are not merely bystanders but active players that can both promote and regulate gut inflammation. Table 1 summarizes key recent studies on eosinophil involvement in IBD across the discussed categories, highlighting their findings and clinical implications.

**Table 1:** Clinical evidences for eosinophil involvement in IBD.

Eosinophil-related parameter	Study (year) & population	Key findings
Peripheral blood eosinophil count	Click et al., (42) (US, *n* = 2066 IBD)	∼19% of IBD patients developed eosinophilia; associated with UC, extensive colitis, and active disease
		– PBE linked to higher CRP, more hospitalizations/surgeries, and faster time to surgery (especially colectomy in UC)
	Yerushalmy-Feler et al. (epi-IIRN), (41)	Prevalence of eosinophilia at diagnosis: 13% in IBD vs. 5% controls (*p* < 0.001); more frequent in UC (16%) than CD (11%), and in pediatric IBD (23%)
	(Israel, *n* = 28,133 IBD)	– Baseline PBE predicted severe disease course (HR ≈ 1.5), including earlier steroid need, biologic initiation, and hospitalization
Tissue eosinophil infiltration	Aulia et al., (45) (Indonesia, *n* = 48 UC)	Moderate positive correlation between colonic eosinophil density and UC severity (*r* = 0.396, *p* = 0.005).
		– Even mild UC had some eosinophils, but severe UC showed markedly higher counts, up to 172 eos/HPF in this study
	Gabriëls et al., (47) (Netherlands, *n* = 84 IBD; subset *n* = 24 biopsies)	**Eosinophils in non-inflamed colon**: Patients who responded to vedolizumab had higher baseline eosinophil counts in *histologically normal* colonic segments (median 69 vs. 24 eos/HPF in non-inflamed tissue, *p* < 0.01).
		– This suggests eosinophil trafficking to gut was active pre-treatment in responders. Baseline serum eotaxin-1 was also higher in responders (0.33 vs. 0.20 ng/ml)
	Haasnoot et al., (43)	In newly diagnosed UC, peak colonic eosinophil counts (median ∼70/HPF) did not correlate with baseline inflammation severity and did not predict 1-year escalation or long-term outcomes
	(Netherlands, *n* = 103 UC)	
Eosinophil activation markers (CD44, CD9, PD-L1)	Lampinen et al., (52)	CD44^high^/CD9^low^ phenotype identified activated eosinophils. Eosinophils are more numerous and active in patients with active ulcerative colitis (UC) compared to controls.
	(Sweden, *n* = 39 UC)	
	Lampinen et al., (55) (Sweden, *n* = 85 IBD)	In active UC/CD, a larger fraction of intestinal eos were CD44^high^ (vs. controls). UC in remission had the *highest* proportion of CD44^high^ eos (median ∼80% vs. ∼50% in active UC), whereas in Crohn's remission eosinophil activation subsided
		– This reflects different cytokine environments: IL-5/IL-13 (prevailing in UC) further increased CD44 on eosinophils, while in CD (more IFN-*γ*), eos activation was lower in remission
	Gurtner et al., (58) (Switzerland, *n* = 5 Crohn; 4 UC)	PD-L1^+^ active eosinophils are enriched in the colonic lamina propria of patients with IBD patients, compared to healthy individuals.
		– These PD-L1^+^ eosinophils are closely associated with CD4^+^ T cells in inflamed tissue, suggesting a role in modulating immune responses during intestinal inflammation.
Eosinophil granule proteins (ECP, EDN, EPX)	Abedin et al., (59) (Germany, *n* = 150 IBD)	**Fecal ECP (fECP)** was elevated in both active UC and CD (mean ∼500–600 µg/kg vs. <50 in controls). fECP had lower diagnostic accuracy than calprotectin for active disease, but was often high even in patients in clinical remission.
		– Notably, among patients with low fecal calprotectin (<250), those with high fECP were much more likely to relapse or require surgery within 4 years
	Amcoff et al., (62) (Sweden, *n* = 104, 2-year follow-up)	**Fecal EDN** (eosinophil-derived neurotoxin): in UC, a rise in EDN often preceded relapse. EDN was significantly increased at flare-up and also detectable at elevated levels 3 months before relapse compared to stable remission
		Each two-fold increase in fecal EDN was associated with ∼30% higher hazard of relapse in UC
		– In Crohn's disease, interestingly, patients who remained in remission had higher baseline EDN than those who relapsed.
	Wędrychowicz et al., (63)	**Serum ECP**: Active IBD patients have elevated serum ECP compared to those in remission
	(Poland, *n* = 125)	– High serum ECP reflects systemic eosinophil activation and correlates with disease activity indices. It normalizes with successful treatment, making it a potential marker for monitoring (though not disease-specific).
	Carlson et al., (64) (Sweden, *n* = 36 IBD)	**EPX/EPO**: Colonic mucosa and luminal fluids in active IBD contain increased eosinophil peroxidase, indicating eosinophil degranulation *in situ*
		– Mucosal EPX was highest at diagnosis and decreased over time in IBD patients
Cytokines & chemokines (Eotaxin, IL-13, CCL5)	Coburn et al., (80)	**Eotaxin-1 (CCL11)**: a potent eosinophil chemokine, with increased levels in both serum and tissue of patients with active UC.
	(USA, *n* = 175)	
	Gabriëls et al., (47)	**Eotaxin-1**: a potent eosinophil chemokine, found elevated in active IBD lesions
	(Netherlands, *n* = 84 IBD; subset *n* = 24 biopsies)	
	Takedomi et al., (79) (Japan, *n* = 83 UC)	**Serum periostin (IL-13 surrogate)** correlates with tissue eosinophils in UC and identifies a subset of “Type 2–high” patients
		– UC patients with high periostin (and hence high IL-13 activity and eosinophil infiltration) achieved higher remission rates on corticosteroids (71% vs. 32% in low-periostin group)
	Seidelin et al., (83)	**IL-33** is upregulated in colonic epithelial cells of patients with UC, with a 13-fold increase in active UC and a 3-fold increase in UC in remission compared to controls.
	(Denmark, *n* = 15 UC)	
	Jeziorska et al, (84)	**RANTES (CCL5)** and eotaxin were upregulated in accumulated eosinophils in active, fulminant inflammatiom tissue
	(UK, *n* = 40 IBD)	

### 3.6 Animal models supporting clinical findings

While clinical investigations reveal correlations between eosinophils and IBD severity, causality is difficult to establish in patients. A number of *in vivo* models of colitis have been developed that replicate the characteristics of IBD (86); these models allow targeted genetic and pharmacologic manipulation of eosinophils, helping to dissect their mechanistic roles. By experimentally depleting eosinophils or altering their recruitment and effector functions in these models, researchers can test if eosinophils are required for inflammation and tissue damage or instead contribute to tissue repair. Below, we summarize key findings from both chemically induced colitis models (e.g., DSS, TNBS) and genetically engineered spontaneous models (IL-10 KO, Winnie, SAMP1/YitFc), each providing mechanistic insights into eosinophil functions and interactions in IBD pathogenesis.

#### 3.6.1 Chemically induced models

• **DSS-induced colitis:** Widely utilized due to reproducibility and acute epithelial injury resembling aspects of ulcerative colitis (87).
  ○ **Eosinophil deficiency (ΔdblGATA mice)** reduced clinical severity, decreased neutrophil infiltration, and improved survival, suggesting eosinophils directly exacerbate DSS colitis (88). In another DSS colitis model, eosinophils are not only recruited to the colon in acute inflammatory stages and but also remain in substantial numbers throughout the mucosal healing process (89). In the microarray analysis of primary colonic eosinophils, s100a8 and s100a9 genes were strikingly increased through healing phase in eosinophil-dependent manner; suggesting protective role of eosinophils *via* induction of s100a8/s100a9.
  ○ **Eosinophil depletion (BM transfer from PHIL mice or IL-5 blockade in WT mice)** leads to worsened colitis and impaired mucosal repair demonstrating protective roles of eosinophils mediated by suppression of excessive neutrophil inflammation and secretion of protective mediators like IL-1Ra and protectin D1 (90).
  ○ **Eosinophil depletion (PHIL mice)** led to increased colitis severity and stronger T helper 17 (Th17) responses, as well as increased production of TNF and IFN*γ* by CD4^+^ T cells (58). In this study, the authors identified a subset of eosinophils (PD-L1^+^ and CD80^+^) with single-cell transcriptomics, which are induced by cytokines such as IL-33 and IFN*γ* and exhibit bactericidal and T cell regulatory functions in DSS-colitis.
  ○ **Eosinophil peroxidase (EPO) deficiency** markedly attenuates DSS colitis, illustrating a pathogenic role for eosinophil granule proteins in driving mucosal damage (67), whereas MBP deficient mice did not show significant changes.
  ○ **CCL11 (eotaxin-1) deficiency** significantly diminishes eosinophil infiltration and improves DSS-induced colitis, affirming the pathogenic role of eosinophil chemotaxis *via* CCL11-CCR3 signaling (91).
  ○ **Blocking eotaxin-1** using a chemokine-binding protein reduced eosinophil accumulation and colitis severity in WT mice (91).
  ○ **CCR2 deficiency or pharmacological inhibition** reduces recruitment of inflammatory Ly6C^hi^ monocytes/macrophages and eosinophils, significantly ameliorating DSS-induced colitis, identifying a key pathogenic role of this monocyte-eosinophil axis *via* CCL11 chemokine production (92).
  ○ **IL-5 deficient mice** show significant reductions in eosinophilic infiltration; however, overall colitis severity remains unchanged, suggesting presence of eosinophils itself may not substantially contribute to pathology in this model (93).
  ○ **Combined IL-2/JES6-1 (IL-2 immunocomplex) and anti-IL-5 treatment** markedly reduces DSS colitis severity by expanding regulatory T cells without eosinophil activation, demonstrating therapeutic potential by uncoupling beneficial Treg expansion from pathogenic eosinophilia (94).
  ○ **Aryl hydrocarbon receptor (AHR) deficient mice** have been demonstrated to manifest exacerbated colitis upon DSS treatment, where AHR, a ligand-activated transcription factor, is known to function as an anti-inflammatory regulator (95). A recent study found increased eosinophil numbers in the small intestine of AHR^−/−^ mice, and eosinophil-specific AHR deletion revealed the tissue adaptation role (cell adhesion, extracellular matrix remodeling) of AHR in eosinophils, suggesting the AHR-mediated capacity of eosinophils to control intestinal inflammation (96).
• **NBS-induced colitis:** Another acute inflammatory model more resemble to Crohn's disease, with mixed innate and adaptive immune responses (97).
  ○ **Immunization with P28GST (schistosome enzymatic protein)** reduced colitis lesions and pro-inflammatory cytokine expression, associated with significant eosinophil infiltration in the colon; eosinophil depletion with anti-Siglec-F treatment or IL-5 knockout abolished the therapeutic benefit, indicating that eosinophils mediate the immuno-regulatory and anti-inflammatory effects of P28GST in colitis (98).
  ○ **Blocking prostaglandin D2 receptor (CRTH2)** significantly reduced eosinophil recruitment and severity of the colitis (99). The protective effect of the CRTH2 antagonist was absent in eosinophil-deficient ΔdblGATA mice.

#### 3.6.2 Spontaneous, genetic, and Microbiota transfer models

• **IL-10 KO mice:** Widely used animal model for studying IBD, especially UC, with spontaneously developed colitis in the presence of intestinal microbiota (100).
  ○ **CCR3 deficiency (CCR3^−/−^;IL-10^−/−^ mice)** results in significantly reduced eosinophilic infiltration but no major change in disease severity or inflammatory cytokine production. This indicates eosinophils may not have a primary pathogenic role in IL-10 deficiency-driven colitis (101).
• **Winnie mice (Muc2 mutation):** Genetic model of chronic epithelial barrier defect-driven ulcerative colitis (102).
  ○ **CCR3 antagonist treatment (SB328437)** substantially decreases eosinophil infiltration and significantly ameliorates colonic inflammation, affirming a pathogenic role for eosinophil-driven chronic inflammation in Winnie colitis (103).
• **SAMP1/YitFc mice:** Genetic model of spontaneous Crohn's-like ileitis (104).
  ○ **IL-33 blockade** profoundly reduces eosinophil infiltration and colitis severity, demonstrating that IL-33–dependent eosinophilia is colitogenic in chronic intestinal inflammation (105).
• **T cell transfer model (IL-23 driven):** Transfer of naive CD4^+^CD45RB^hi^ T cells into immunodeficient mice results in IL-23-dependent colonic inflammation which mimics the T cell-driven immune response against intestinal antigens observed in human IBD (106).
  ○ **GM-CSF activated eosinophils** enhance IL-23 driven chronic colitis. Neutralizing GM-CSF or depleting eosinophils significantly reduces colitis severity, directly demonstrating eosinophil pathogenicity in chronic adaptive immune-driven colitis (107).

In summary, animal models support a nuanced, context-dependent role for eosinophils in IBD. Acute models (DSS/TNBS) show eosinophils can either mediate mucosal damage via granule proteins and pro-inflammatory cytokines or, paradoxically, limit excessive inflammation by regulatory mediators. In chronic models (SAMP1/YitFc, Winnie, IL-23-driven T cell transfer), eosinophils more consistently drive tissue pathology through cytokines like IL-33, IL-5, GM-CSF, and chemokines like CCL11 (Summarized in Table 2).

**Table 2:** Summary of animal studies investigating eosinophil roles in IBD.

IBD model	Intervention (genetic/pharmacological)	Phase	Key result	Suggested role of eosinophils	References
DSS	CCR2 KO (monocyte recruitment)	Acute	Reduced Ly6C^hi^ monocytes, eosinophils, and severity	Colitogenic	Waddell, (92)
DSS	Eosinophil deficiency (ΔdblGATA mice or CCL11 blockade)	Acute	Reduced tissue injury	Colitogenic	Vieira et al., (88)
DSS	Eosinophil deficiency (ΔdblGATA)	Acute	Reduced severity/Decreased s100A/s100B expression in healing phase	Colitogenic	Reichman et al., (89)
DSS	Eosinophil depletion (BM transfer from PHIL mice or IL-5 blockade)	Acute	More severe colitis	Protective	Masterson et al., (90)
DSS	Eosinophil depletion (PHIL mice)	Acute	Increased colitis severity and stronger T helper 17 (Th17) responses, production of TNF and IFNγ by CD4^+^ T cells.	Protective	Gurtner et al., (58)
DSS	EPO^−/−^ mice (EPX knockout)	Acute	Reduced tissue injury	Colitogenic	Forbes et al., 2004 (65)
DSS	*Ccl11*^−/−^ mice (Eotaxin-1 deficiency)	Acute	Reduced eosinophil infiltration and inflammation	Colitogenic	Polosukhina et al., (91)
DSS	IL-2/JES6-1 + anti-IL-5 mAb	Acute	Expanded Treg cells without eosinophilia, significantly ameliorated colitis	Colitogenic	Abo et al., (94)
DSS	IL-5 knockout	Acute	Reduced eosinophil numbers but no significant change in severity	Non-essential	Stevceva et al., (93)
DSS	AHR deficiency	Acute	Increased eosinophil infiltration and inflammation	Colitogenic without AHR-mediated regulation	Diny et al., (96)
TNBS	CRTH2 blockade or eosinophil deficiency (ΔdblGATA mice)	Acute	Reduced eosinophil infiltration and inflammation	Colitogenic	Radnai et al., (99)
TNBS	Immunization with P28GST and eosinophil depletion (IL-5KO or anti-Siglec-F)	Acute	Depletion of eosinophils abolished the anti-inflammatory effects of P28GST.	Protective	Driss et al., (98)
IL-10 KO	CCR3 knockout	Chronic	Reduced eosinophil infiltration without affecting severity	Non-essential	Wang et al., (101)
Winnie	CCR3 antagonist (SB328437)	Chronic	Reduced eosinophils and significantly attenuated colitis severity	Colitogenic	Filippone et al., (103)
SAMP1/YitFc	IL-33 blockade	Chronic	Reduced eosinophils and ileitis severity	Colitogenic	Salvo et al., (105)
T cell transfer (IL-23 driven)	GM-CSF/eosinophil blockade	Chronic	Reduced eosinophils and significantly attenuated chronic colitis	Colitogenic	Griseri et al., (107)

Collectively, substantial evidence links eosinophils with IBD activity: they are often increased in blood and gut during flares, carry activation markers, release toxic granule contents correlated with disease, and engage in crosstalk with the type 2 immune axis (IL-5, eotaxin). These findings span histological observations from the mid-20th century to recent clinical correlations and complementary animal studies, collectively supporting that eosinophils are not innocent bystanders but actively implicated in IBD pathogenesis and contributing to post-inflammatory remodeling.

## 4 Model of ROS-mediated intestinal homeostasis and eosinophil-derived ROS

The exact conditions under which eosinophils harm or heal the gut are an area of active investigation. The following sections delve into an integrative model that may explain how eosinophils can have such paradoxical effects. Central to this model is ROS—reactive oxygen species—as both effectors and regulators of inflammation, and Siglec-8–sialic acid interactions as a molecular brake on eosinophil activity. Understanding these could illuminate why eosinophils sometimes exacerbate IBD and how we might therapeutically modulate them.

It is now appreciated that appropriate levels of reactive oxygen species (ROS) in the gut mucosa are crucial for host defense and tissue homeostasis. Historically, ROS (such as superoxide and hydrogen peroxide) were considered purely deleterious, causing oxidative stress and cell damage (108). Indeed, excessive ROS production is implicated in tissue injury in IBD—mucosal biopsies from active IBD show increased markers of oxidative damage (109), and patients often have elevated levels of ROS byproducts (110, 111). However, a paradigm shift has occurred with the recognition that physiological ROS signaling is essential for normal intestinal function (2, 112). ROS act as signaling molecules that regulate epithelial proliferation (113), mucus production (114), and immune responses (115, 116). The intestinal epithelium and some commensal bacteria constitutively generate low levels of ROS as part of the homeostatic milieu (117–119). Key sources of ROS in the gut include NADPH oxidase enzymes (120). Epithelial cells express NOX1 and DUOX2, which produce ROS into the extracellular space or gut lumen (121–123), whereas phagocytes (neutrophils, eosinophils, macrophages) express NOX2 (phagocyte oxidase) that produces superoxide inside phagosomes or can leak it outside (124, 125). These ROS have antimicrobial effects (helping to kill ingested microbes) and also modulate signaling pathways that influence barrier function. For example, NOX1-derived ROS in epithelial cells have been shown to promote wound healing responses—ROS can act as second messengers to activate proliferative signaling and cytoprotective genes in epithelial cells (126, 127). Controlled ROS can induce antioxidant defenses and fortify the epithelium (128). ROS's protective role was directly demonstrated in a study, for example, that showed that mice with genetic ablation of NOX1 had impaired mucosal repair following DSS-induced colitis (129). Such findings suggest that inadequate ROS production impairs mucosal immunity, allowing pathogenic bacteria to persist and leading to chronic inflammation. In line with this, human genetic studies have found that loss-of-function mutations in ROS-producing enzymes predispose to IBD: patients (especially with very-early-onset IBD) carrying defective NOX2 (as in chronic granulomatous disease) or mutations in NOX1/DUOX2 often develop colitis (130–132). This is a striking inversion of the old idea—too little ROS can be as problematic as too much, because ROS are needed to keep microbiota in check and to signal tissue repair.

In the context of this ROS paradigm, eosinophils emerge as important contributors to the ROS landscape of the intestine. Eosinophils are particularly potent producers of hydrogen peroxide (H_2_O_2_) and other ROS (133, 134). Upon activation, eosinophils undergo a “respiratory burst” much like neutrophils, via the NADPH oxidase complex (of note, eosinophils highly express gp91^phox^ and other subunits of NOX2) (127, 135, 136). Crucially, eosinophils excel in generating extracellular ROS in the forms of respiratory burst, a capacity that vastly surpasses that of neutrophils (137, 138). Eosinophil peroxidase (EPX) then uses H_2_O_2_ to generate hypobromous acid and other oxidants (139). Thus, eosinophil-derived ROS are abundant in IBD lesions and likely contribute to the oxidative injury observed in mucosal tissues. These ROS can directly damage epithelial cells and extracellular matrix, leading to ulceration and propagating inflammation.

**Figure 1:**
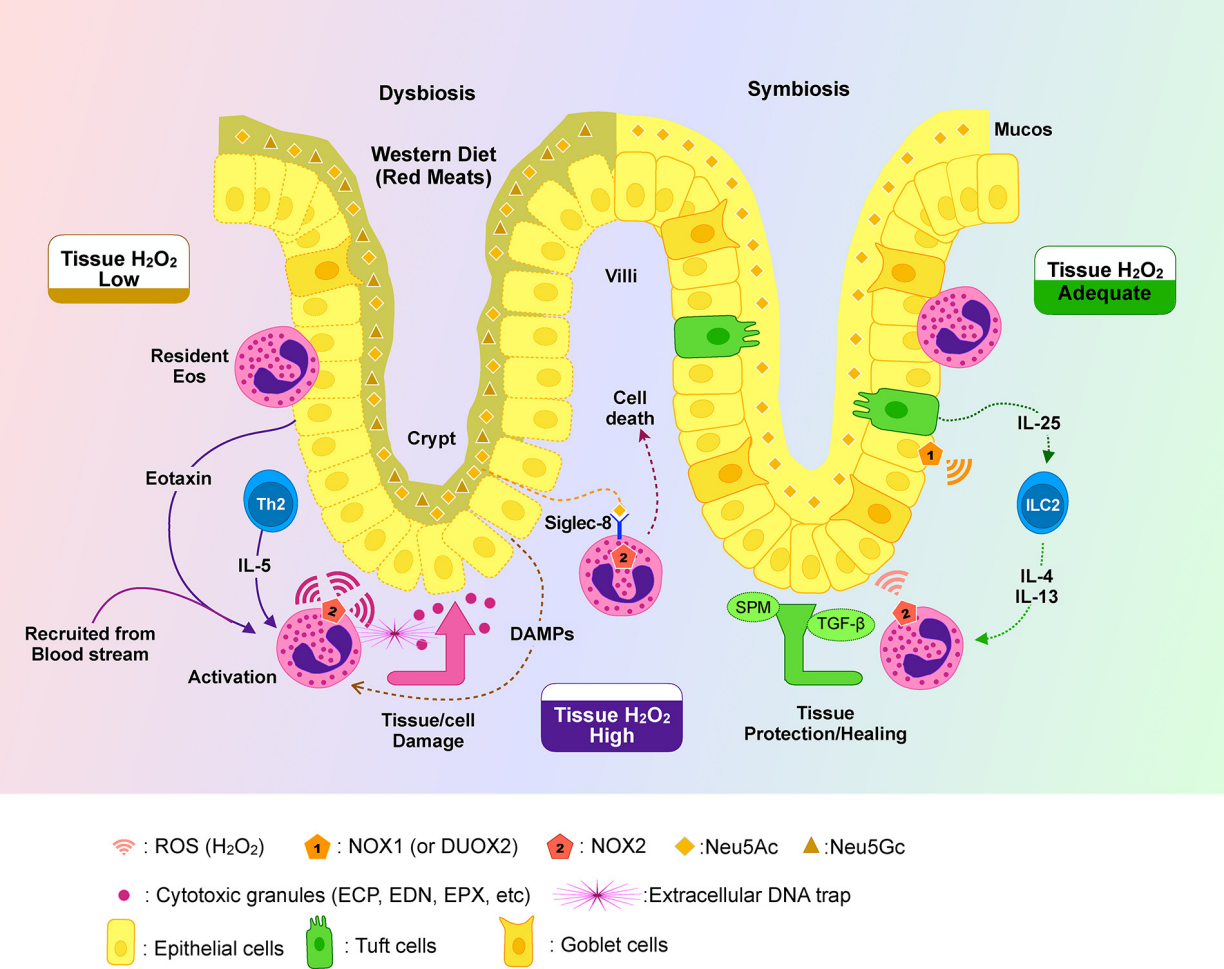
Proposed model of ROS-mediated intestinal homeostasis and the roles of Eos, Siglec-8 and sialic acids. Eosinophils are recruited from the bloodstream into the intestinal mucosa via epithelial-derived Eotaxin, where they reside mainly in the villus and crypt lamina propria. Upon activation by IL-5 and local DAMPs, eosinophils release cytotoxic granules (ECP, EDN, EPX), ROS (especially H₂O₂), and extracellular DNA traps to combat pathogens and potentially injure tissue. Neu5Ac-containing sialic acids released from damaged tissue engage Siglec-8, inducing eosinophil apoptosis and limiting excessive inflammation. However, Western diets rich in Neu5Gc may impair this checkpoint due to insufficient activation of Siglec-8-mediated anti-inflammatory process. Tuft cell-derived IL-25 activates ILC2s, promoting IL-4 and IL-13 production that further modulates eosinophil function. Adequate ROS levels support epithelial repair via NOX1/DUOX2, but dysbiosis or NOX mutations may disrupt this balance, skewing tissue responses toward chronic inflammation.

Yet, eosinophil ROS may also have beneficial roles. In physiological conditions, eosinophils patrol the lamina propria and could provide a basal oxidative tone that supports host-microbe homeostasis (26, 140). Eosinophils can kill invasive microorganisms (bacteria, fungi) via ROS, protecting against infection (141, 142). Although there is no direct evidence proving eosinophils uses low level ROS to induce anti-inflammatory/healing process, it is noteworthy that eosinophils command a more diverse arsenal of reactive oxygen species (ROS) sources compared to the highly specialized neutrophil. While both cells utilize NADPH oxidase 2 (NOX2) for potent oxidative bursts, eosinophils are uniquely enriched with abundant mitochondria for sustained, low-level ROS production and are known to express other isoforms like NOX1 and DUOX enzymes (143–146). As described above, eosinophil-deficient mice (ΔdblGATA mice) have been shown to exhibit delayed recovery from acute colitis in some studies, possibly due to the loss of eosinophil-derived healing factors, which may include ROS and ROS-induced signals (though results vary across models). Figure 1 (proposed model) illustrates this delicate balance: an “adequate” tissue ROS level contributes to tissue protection and healing, whereas either extreme—too high ROS causing cell damage, or too low ROS failing to control microbes and stimulate repair—leads to pathology.

Indeed, cell and animal studies now suggest that maintaining the right amount of ROS in tissues is key to intestinal homeostasis, and eosinophils are integral to this balance. During active inflammation (e.g., an infection or flare of IBD), eosinophils are recruited by chemokines like eotaxin to the gut mucosa^1^. In their activated state (for instance, stimulated by IL-5 or by alarmins like IL-33 and IL-25), eosinophils unleash an arsenal that includes ROS along with cytotoxic granule proteins and cytokines (147, 148). This helps contain invading pathogens and digest necrotic tissue—a necessary defense. However, collateral damage is a risk: ROS and granule proteins can injure host cells, perpetuating inflammation. This is where the concept of a built-in braking mechanism becomes critical (discussed in the next section). Conversely, in the resolution phase, eosinophils are thought to contribute to tissue repair: they can undergo “partial activation” that favors release of growth and remodeling factors (e.g., VEGF, TGF-β) (149–152). Notably, eosinophil-secreted TGF-β has been observed to stimulate environmental cells to produce fibronectin, a protein crucial for wound healing and tissue regeneration that is found in the extracellular matrix (153). Intriguingly, fibronectin has in turn been shown to induce low-level ROS production in eosinophils (154), which may act as signals for restitution rather than causing injury.

As described earlier, eosinophils interacting with type 2 innate lymphoid cells (ILC2s) and tuft cell-derived IL-25 may shift into a pro-healing phenotype. Tuft cells in the gut epithelium continuously secrete IL-25 that helps maintain ILC2 activity. In response, ILC2s produce IL-5 and IL-13, which not only recruit eosinophils but also modulate their function to promote epithelial regeneration (29, 30). This cooperative circuit—tuft cells → IL-25 → ILC2 → IL-5/IL-13 → eosinophils—is crucial in expelling intestinal helminths, but evidence suggests it also underlies tissue adaptation to injury (even in non-parasitic settings) (155, 156). In the context of ROS, Tuft cells have succinate receptor 1 (SUCNR1/GPR91) and are prompted to initiate type 2 innate immune circuit by succinate (157–159), which is known to increase under stress and in high ROS situations (160, 161), and has also been found to promote mitochondrial ROS production (162, 163). Elevated levels of succinate in the gut are a known feature of IBD and other conditions of intestinal inflammation and microbial imbalance (dysbiosis) (164, 165), as evidenced by elevated serum and intestinal succinate levels and SUCNR1 expression in Crohn's disease patients (166). Thus, in IBD, it is plausible that this type 2 circuit tries to counteract the damage, with eosinophils at the center of repair signals. However, if the epithelial or microbial environment is abnormal (as in chronic dysbiosis or in presence of ROS-blocking mutations), this homeostatic ROS mechanism may falter.

A striking piece linking dysbiosis and ROS comes from metabolomic observations: some dysbiotic microbial communities in IBD show enhanced glycolytic metabolism at the expense of oxidative pathways, potentially yielding less H_2_O_2_ in the gut (167–169). Commensal bacteria such as certain Lactobacillus species normally produce peroxide as a metabolic byproduct that can inhibit competing microbes and modulate immune cells (170, 171). If dysbiosis leads to fewer such microbes or altered metabolism, the local ROS milieu might be deficient, impairing the triggers for mucosal healing. Additionally, IBD-associated genetic variants in NOX1, NOX2, DUOX2 and related regulators (e.g., CYBA) underscore that a failure to generate ROS predisposes to uncontrolled inflammation (172, 173). In this scenario, eosinophils arriving to repair the tissue might overcompensate by releasing large amounts of ROS (since baseline signaling is absent)—ironically causing more harm. Or, eosinophils might not receive the proper ROS cues to transition into a pro-resolving phenotype, thereby remaining in an inflammatory state longer.

In summary, we propose a model in which eosinophil-derived ROS serve as a central mediator of intestinal homeostasis, performing a dual role. On one hand, ROS from eosinophils (and epithelial cells) at appropriate levels promote epithelial growth, mucus secretion, and pathogen control—essential processes for maintaining mucosal integrity. On the other hand, excess ROS release (e.g., during an uncontrolled eosinophil activation in a flare) causes tissue damage and amplifies inflammation. Thus, tight regulation of eosinophil activation and ROS production is necessary. The body appears to have evolved specific mechanisms for this regulation—one of which involves Siglec-8 and sialic acids as a negative feedback pathway to turn off eosinophils when they start causing tissue injury. Other potential candidates for the regulator may include eosinophil-derived tissue repairing factors, such as SPM or amphiregulin (174), but the non-specificity of these factors to eosinophils makes them less compelling as major conductors of eosinophil behavior. In the next section, we delve into how Siglec-8 functions as a brake on eosinophils and how its interaction with sialic acid ligands (Neu5Ac vs. Neu5Gc) might be influenced by diet and disease in IBD contexts.

## 5 Siglec-8 and sialic acid-mediated ROS regulation in eosinophils

To prevent immune cells from inflicting excessive damage, the body employs inhibitory receptors that act as checks and balances. In eosinophils, one such key inhibitory receptor is Siglec-8. Siglec-8 (Sialic acid-binding Ig-like lectin 8) is uniquely expressed on human eosinophils and mast cells (with low levels on basophils) (4, 175, 176). It belongs to the CD33-related Siglec family which contains immunoreceptor tyrosine-based inhibitory motifs (ITIMs) in its cytoplasmic tail and recognize sialic acids on host cells as self-associated molecular patterns (SAMPs) to dampen innate immune responses (177, 178). When Siglec-8 is engaged (for example, by antibodies or its natural glycan ligands), it delivers a potent negative signal: in eosinophils this triggers apoptosis (179), and in mast cells it inhibits degranulation (180). Functionally, Siglec-8 acts as an “emergency off-switch” for eosinophils—a way to induce eosinophil cell death and stop their release of inflammatory mediators when needed.

The discovery of Siglec-8's effect was first made by cross-linking Siglec-8 with antibodies, which caused rapid eosinophil apoptosis *in vitro* (181). Importantly, this apoptosis was found to be enhanced if eosinophils were in an activated state (primed by cytokines). This is somewhat counterintuitive: typically IL-5 and GM-CSF prolong eosinophil survival, but in the presence of Siglec-8 engagement, IL-5 “priming” makes eosinophils die faster. Nutku et al. demonstrated that eosinophils cultured with IL-5 became more susceptible to Siglec-8-mediated cell death, and the character of that cell death changed—it no longer relied on the classical caspase pathway but instead depended on mitochondrial damage and ROS generation within the eosinophil (182). Specifically, IL-5 priming led Siglec-8 cross-linking to cause a loss of mitochondrial membrane potential and production of ROS inside the eosinophil, which in turn drove the cell into apoptosis through a caspase-independent mechanism (183). Inhibitors of NADPH oxidase and mitochondrial respiration could block this apoptosis, confirming that an oxidative burst within the eosinophil is a key part of Siglec-8 signaling after IL-5 priming. These findings are fascinating because they imply a feedback loop: an activated eosinophil (full of ROS and cytotoxic granules) is prompted by Siglec-8 to essentially self-destruct via its own ROS, thereby preventing it from causing unchecked tissue damage. From a clinical perspective, it suggests that in disease states with high IL-5 (like hypereosinophilic syndromes or active IBD with a type 2 skew), targeting Siglec-8 could be especially effective in depleting pathogenic eosinophils.

So what engages Siglec-8 *in vivo*? The natural ligands of Siglec-8 are complex sialoglycans—sugars containing sialic acid in specific linkages and modifications. Glycan array studies show Siglec-8 is highly selective: it prefers sialic acid linked α2-3 to galactose, where that galactose carries a sulfate group at the 6-position [i.e., 6′-sulfo-sialyl Lewis X structure, Neu5Acα2-3Gal(6S)β1-4GlcNAc…] (184, 185). In particular, Siglec-8 recognizes keratan sulfate chains terminating in 6-sulfo-sialyl LacNAc (186). Endogenous ligands for Siglec-8 have been identified in human airways—notably on aggrecan in cartilage and on secreted mucus glycoproteins in submucosal glands (187, 188). These ligands carry the requisite sialylated, sulfated motifs and their binding to Siglec-8 on eosinophils can induce apoptosis. In the gut, the specific Siglec-8 ligand distribution is less characterized, but colonic mucus and epithelial glycocalyx are rich in sialylated and sulfated glycans that could serve as ligands (189). There is evidence that injury or stress in tissues leads to the exposure or release of Siglec ligands, which then engage receptors like Siglec-8 on infiltrating cells (181). In an IBD context, one could speculate that during epithelial damage, sialylated glycoepitopes (especially those containing Neu5Ac) are exposed or shed, and eosinophils encountering these in the lamina propria via Siglec-8 may be signaled to undergo apoptosis. This would constitute a negative feedback loop to halt eosinophil-driven inflammation once sufficient tissue damage has occurred (i.e., “don't overshoot the response”).

However, a intriguing twist lies in the type of sialic acid. Sialic acids come in different forms, primarily N-acetylneuraminic acid (Neu5Ac) and N-glycolylneuraminic acid (Neu5Gc) (190). Humans, due to a mutation in the CMAH gene, cannot synthesize Neu5Gc and predominantly use Neu5Ac on our cell surfaces (191). Neu5Gc is present in other mammals and can be obtained through diet (red meat is rich in Neu5Gc) (192); when humans ingest Neu5Gc, small amounts can incorporate into our tissues (193, 194). Siglecs evolved under different sialic acid environments and often show preferences: some Siglecs bind Neu5Gc better, others prefer Neu5Ac. For example, murine Siglec-1 strongly prefers Neu5Ac, whereas some primate Siglecs have affinity for Neu5Gc (195, 196). In the case of Siglec-8, its known ligand from human studies contains Neu5Ac (the 6′-sulfo-sialyl Lewis X has Neu5Ac) (197). It's not explicitly reported to prefer Neu5Gc, and given human Siglec-8 evolved in a Neu5Ac-dominated environment, it likely requires Neu5Ac in its binding motif.

This brings up an important hypothesis: could a high incorporation of Neu5Gc into colonic tissue (*via* a Western diet high in red meat) impair Siglec-8's ability to recognize its ligands, thus blunting the “brake” on eosinophils? Figure 1 posits exactly that—Western diet increases Neu5Gc content in tissues, which might render Siglec-8 less effective at sensing tissue damage. In other words, if epithelial or extracellular matrix glycans bear Neu5Gc instead of Neu5Ac, Siglec-8 on eosinophils may not bind well, and eosinophils would not receive the apoptosis signal even when damage is occurring. As a result, eosinophils could continue to release ROS and granules unabated, causing excessive inflammation. This hypothesis is supported indirectly by knowledge that some Siglecs lose binding when Neu5Gc is present. For instance, human Siglec-9 and Siglec-7 binding can differ with Neu5Ac vs. Neu5Gc, and generally, the absence of Neu5Gc in humans has altered Siglec biology compared to other species (198, 199). Additionally, Neu5Gc in human tissues is immunogenic (people develop anti-Neu5Gc antibodies), which can cause chronic inflammation—potentially another link between red meat diets and colitis severity (though speculative) (200). While direct evidence in IBD is lacking, it's an intriguing area: dietary sialic acids could modulate immune regulation. It suggests that dietary interventions to reduce Neu5Gc (e.g., limiting red meat) might theoretically enhance Siglec-8's protective signaling, whereas Neu5Gc-rich diets might worsen eosinophil-driven inflammation by undermining this checkpoint (201–203).

It is noteworthy that humans are unique in having Siglec-8; mice do not have a direct Siglec-8 ortholog. Mice express Siglec-F on eosinophils, which is often cited as a functional analog. Siglec-F also induces eosinophil apoptosis in mice and regulates eosinophilic responses in the lung (204). However, Siglec-F and Siglec-8 have differences in ligand specificity and expression. For example, Siglec-F recognizes similar 6-sulfated sialyl glycans but also some different glycans, and it binds to some human airway ligands that Siglec-8 does not (197). This species difference complicates direct translation of mouse findings to human IBD, but it reinforces that the Siglec-eosinophil inhibitory pathway is evolutionarily important in controlling eosinophils. In mouse colitis models, engaging Siglec-F (such as with a Siglec-F antibody) leads to eosinophil apoptosis and has been shown to reduce colonic eosinophil numbers, although the impact on inflammation severity varied by model (some acute colitis models didn't improve, whereas chronic models or asthma models clearly benefited from eosinophil suppression) (205–208).

In the gut, what could be the natural trigger for Siglec-8 engagement? We hypothesize two scenarios: (1) Tissue damage release of ligands—during epithelial ulceration or cell turnover, membrane fragments rich in sialylated glycoproteins could be shed from mucus layers. Many of these have Neu5Ac termini that could engage Siglec-8 on incoming eosinophils. For instance, high-molecular-weight glycoproteins from goblet cell mucins or enterocyte surfaces might carry the appropriate motif. (2) Counter-receptor expression on other cells—certain endothelial or epithelial cells might express Siglec-8 ligands on their surface in response to cytokines. In fact, the expression of ligands for Siglec-8 (and Siglec-9) is significantly increased in the context of inflammation in human airways, particularly in cases of chronic rhinosinusitis, which is dependent on enhanced biosynthesis by sialyltransferase (209). There is precedent in other systems: inflammatory stimuli upregulate some sialyltransferases and can increase the sialylated glycan ligands for Siglecs (like CD22 ligands or Siglec-1 ligands on endothelium) (210–212). In an inflamed colon, perhaps cytokines like IL-13 or IL-22 modulate glycosylation patterns, increasing 6-sulfation of mucins (thereby creating Siglec-8 ligand epitopes). This is speculative but testable by glycomic analysis of IBD tissues vs. healthy intestinal tissues.

One more intriguing result from Kano et al. (and others) is that Siglec-8 signaling involves ROS generation within eosinophils (193, 213). It appears Siglec-8 engagement in IL-5-primed eosinophils triggers ROS production dependent on NADPH oxidase (and mitochondria in some degree), leading to apoptosis accompanied by the release of eosinophil granules such as EPX. Although this NADPH oxidase-dependent eosinophil cell death is mechanistically similar to EETosis, a crucial difference is that Siglec-8/IL-5 co-stimulation does not elicit an extracellular oxidative burst (214). The mechanism underlying this discrepancy remains unclear, but the author's previous studies suggest differential surface expression of NADPH oxidase subunits might be involved (215). If this internal ROS burst is blocked (as by high antioxidants or perhaps in certain metabolic states), Siglec-8-induced death might be less effective. There may be crosstalk with other eosinophil inhibitory pathways too, such as CD300a (216), but Siglec-8 is unique in its potent, eosinophil-specific action.

In sum, Siglec-8 serves as a critical checkpoint on eosinophils, particularly relevant in settings of chronic inflammation like IBD where eosinophils are abundant. It senses sialylated “self” signals that likely indicate tissue integrity is being threatened. When activated, Siglec-8 curtails eosinophil survival and function—thereby regulating ROS output and preventing excessive collateral damage. Factors like IL-5 paradoxically enhance Siglec-8's pro-apoptotic signaling, making this pathway especially significant when eosinophils are activated in a type 2 cytokine milieu. Meanwhile, the efficacy of this pathway can potentially be modulated by the biochemical nature of sialic acids present (Neu5Ac vs. Neu5Gc)—raising the possibility that diet and metabolic state influence eosinophil regulation. This provides a mechanistic link between environmental factors (e.g., Western diet) and the control of inflammation at the level of innate immune cells; the overall picture of proposed model is demonstrated in Figure 1.

Having outlined this ROS-Siglec-8 model, we next consider what it implies for future research and therapy. Can we harness Siglec-8 or mimic its ligands to treat IBD? How might we restore the ROS balance in the gut? We explore these questions in the final section.

## 6 Future directions and therapeutic implications

The ROS-eosinophil-Siglec-8 hypothesis for IBD pathogenesis offers several avenues for further investigation and intervention. While it presents an appealing unifying model, it is not without limitations and unanswered questions. Here, we outline key future directions:

1. **Validating the ROS Homeostasis Model**: Future studies should directly test whether augmenting controlled ROS levels can protect against IBD or promote healing. For example, in animal models, could enhancing epithelial NADPH oxidase activity (e.g., by administering low levels of ROS donors or gene therapy to boost NOX1/DUOX2 function) reduce colitis severity? Conversely, targeted deletion of ROS sources specifically in eosinophils (using eosinophil-specific NOX2 knockout mice) would clarify how much eosinophil-derived ROS contributes to both injury and repair. Preliminary evidence suggests protective roles for ROS in gut homeostasis (134), but translating that to therapy is tricky—systemic antioxidants mostly failed in IBD trials, perhaps because they also block beneficial ROS (217, 218). A more nuanced approach might involve spatiotemporal control of ROS: delivering ROS-scavenging agents only during flares to limit damage, and conversely providing pro-oxidant stimuli during remission to bolster barrier function. Additionally, mapping ROS gradients in the gut (with novel redox-sensitive probes) could identify whether there is indeed a ROS “deficiency” in dysbiotic or genetically susceptible intestines.
2. **Eosinophil Subpopulations and Siglec-8 Dynamics**: Not all eosinophils are equal—as mentioned, there may be inflammatory vs. regulatory eosinophil subsets (distinguished by surface markers, granule content, or cytokine production) (58, 219, 220). Future single-cell RNA sequencing of colonic eosinophils from IBD patients could reveal distinct states, such as a pro-inflammatory state (high IL-1β, TNF, oxidative burst genes) and a pro-resolution state (high TGF-β, IL-10, ALOX15 for lipid mediators). Understanding how Siglec-8 expression or responsiveness varies between these states is crucial. It's possible that in highly activated eosinophils, Siglec-8 is upregulated as a feedback (some receptors increase upon activation). Or chronic inflammation might downregulate Siglec-8, rendering eosinophils “unchecked.” Flow cytometric analysis of Siglec-8 levels on blood and intestinal eosinophils in IBD could address this. Similarly, examining Siglec-8 ligand expression in colon tissues (using Siglec-8-Fc chimera staining) in IBD vs. healthy controls could identify deficits in ligand availability. If Neu5Gc incorporation is an issue, mass spectrometry of sialic acids in colonic biopsies might correlate Neu5Gc levels with eosinophil activity or disease severity.
3. **Dietary Interventions and Microbiota**: The hypothesis that a Western diet (rich in red meat Neu5Gc) impairs eosinophil regulation *via* Siglec-8 can be explored epidemiologically and experimentally. Epidemiologically, one could examine IBD patient cohorts for correlations between red meat intake, tissue Neu5Gc (perhaps inferred by anti-Neu5Gc antibody titers) (221), and markers of eosinophil activation or disease outcomes. If a link is found, a dietary trial reducing Neu5Gc intake in IBD patients could see if eosinophil counts or IBD activity are impacted. Indeed, dietary interventions that may curtail Neu5Gc have already been implemented for IBD, including the Mediterranean diet, the anti-inflammatory diet for IBD (IBD-AID), and the Crohn's disease exclusion diet (CDED) (222–224). Experimentally, humanized mice that express Siglec-8 and fed with Neu5Gc-rich vs. Neu5Gc-free diets could be subjected to colitis to see if outcomes differ. Another angle is the microbiome: some gut bacteria can cleave sialic acids from host glycans. In dysbiosis, altered sialidase activity might expose more or fewer Siglec-8 ligands. CDED and other dietary treatments for IBD, including exclusive enteral nutrition and the Specific Carbohydrate Diet (SCD), are known to affect the microbiome, which may reduce the abundance of bacteria that produce sialidase enzymes (225–227). Fecal microbiota transplant (FMT) experiments could test if restoring a healthy microbiome (with presumably normal ROS production and glycosidase balance) can normalize eosinophil behavior in colitis models.
4. **Therapeutic Targeting of Eosinophils in IBD**: Given the evidence of eosinophils' involvement, therapies aimed at eosinophils warrant consideration:
  • **Siglec-8 Agonists**: Perhaps the most direct translation of this review's discussion is using Siglec-8 targeting drugs. A monoclonal antibody against Siglec-8 (lirentelimab, AK002) has been developed and is in clinical trials for eosinophilic gastritis and other eosinophil-driven diseases. It triggers eosinophil apoptosis and has shown efficacy in reducing eosinophil numbers and symptoms in eosinophilic GI disorders (228), although in the phase III trial, lirentelimab did not sufficiently improve symptoms (229). For IBD, especially UC, a Siglec-8 agonist could theoretically reduce mucosal eosinophils and their inflammatory products. One advantage is that Siglec-8 is fairly specific; unlike broad IL-5 or IL-5R blockade (which also reduce eosinophils but affect basophils somewhat and systemic eosinophils), Siglec-8 mAb would act on tissue eosinophils and mast cells without markedly suppressing other immune cells. However, caution is needed—completely removing eosinophils might impair the healing aspects they contribute. Perhaps an ideal use is short-term Siglec-8 engagement during acute flares to prevent tissue injury, then allow eosinophils to repopulate for repair.
  • **Chemokine/cytokine Inhibitors**: Blocking eosinophil recruitment with CCR3 antagonists or anti-eotaxin antibodies could reduce eosinophil infiltration into the gut. Some small molecules targeting CCR3 were developed for asthma (230, 231); they could be repurposed for IBD if eosinophil-driven pathology is confirmed. Reducing recruitment might be gentler than killing eosinophils, and could be combined with other anti-inflammatory treatments. Another possibility is the use of dupilumab (IL-4/IL-13 receptor blocker), which has shown promising results in the treatment of various eosinophil-associated diseases including atopic dermatitis, asthma, nasal polyps, and eosinophilic esophagitis (232, 233). Reports on the use of dupilumab for IBD are currently limited, with a report showed no worsening of IBD when dupilumab was used in patients with atopic dermatitis who also had IBD (234). Dupilumab may cause transient eosinophilia, which requires caution (235).
  • **Modulating ROS Pathways**: If one accepts that boosting controlled ROS is beneficial, paradoxically low-dose pro-oxidants or NOX activators could be explored. For instance, there are experimental compounds that activate Nrf2 (and in doing so, sometimes increase baseline antioxidant responses and possibly ROS signaling) (236). Another approach is microbial therapies: introducing or encouraging commensals that produce H_2_O_2_ (such as certain lactic acid bacteria) could elevate mucosal ROS to protective levels. Probiotics might be engineered to secrete low levels of H_2_O_2_ or SPMs that recruit eosinophils into a healing phenotype.
    Various animal models demonstrates that FMT effectively mitigates intestinal oxidative stress by lowering the production of ROS (237). This is achieved by reducing inflammatory triggers like lipopolysaccharides, which in turn decreases ROS-induced damage, as indicated by lower levels of malondialdehyde and a corresponding increase in the activity of host antioxidant enzymes, including catalase, superoxide dismutase, and glutathione peroxidase (238). While direct evidence from human clinical trials is currently limited, a study on donor selection for FMT in UC revealed that successful FMT donor microbiomes exhibited higher oxygen tolerance, indicating the importance of facultative anaerobes in re-establishing an anaerobic environment to counteract ROS (239). Notably, the study also linked the catabolism of sialic acid to unsuccessful FMT outcomes, suggesting that its degradation by certain microbes can contribute the exacerbated inflammation.
  • **Glycan-based therapies**: If specific sialylated glycans can engage Siglec-8, one could design a therapy using a synthetic Siglec-8 ligand. For example, a stabilized 6′-sulfo-sialyl-LacNAc polymer could be administered orally (if it can reach the colon) or intravenously to bind Siglec-8 on eosinophils and temper them. This might avoid some complexities of antibodies and also could be tuned to not completely ablate eosinophils (perhaps delivering a milder signal). Nanoparticles coated with Siglec-8 ligands are another potential strategy to target eosinophils in tissues.
  • **Eosinophil-mediated wound healing enhancement**: On the flip side, perhaps we can harness the good side of eosinophils. For patients with refractory ulcers (as sometimes seen in Crohn's), one might actually want eosinophil activity for healing. Perhaps locally applying IL-5 or IL-33 to an ulcer bed could attract eosinophils to promote fibrosis and closure (risky in terms of inflammation, but conceptually interesting). This is speculative and likely not a near-term approach, but underscores that eosinophils are a tool the body uses for remodeling tissue.
5. **Integrating Allergic and IBD Paradigms**: IBD and allergic diseases (like asthma or food allergy) were traditionally seen as separate, one Th1/Th17-dominant, the other Th2-dominant. Eosinophils and Siglec-8 sit at an intersection—they are classically allergy-related, but clearly relevant in IBD as well. An emerging clinical observation is some overlap of eosinophilic GI disorders (EGIDs) with IBD; e.g., patients with eosinophilic esophagitis have a higher risk of developing IBD (240). It prompts the question: is there a common pathway of epithelial barrier defect and type 2 inflammation that underlies both? Future research might look at patients who have features of both EGID and IBD (“overlap colitis”) to see if they represent a distinct endotype characterized by eosinophil dysfunction. These patients might especially benefit from therapies like anti-IL-5 or anti-Siglec-8. Additionally, studying Siglec-8 in allergy vs. in IBD could reveal differences: for instance, in asthma, lung epithelial Siglec-8 ligands are upregulated and may naturally limit eosinophilic inflammation (241); do colitis patients fail to upregulate colonic Siglec-8 ligands? That could be a key pathogenic difference.
6. **Safety of Targeting Eosinophils**: While eosinophils can be pathogenic, they are also part of normal immunity. Completely removing eosinophils (as done in some trials with IL-5 antibodies) surprisingly doesn't cause major immunodeficiency in adults, but subtle effects exist (e.g., delayed clearance of some viruses, possible impact on tissue remodeling) (242). Long-term depletion might affect cancer surveillance or other processes (243). Recently, it has become widely accepted that tumor cells may coat themselves with sialic acid to induce immune escape *via* engagement with several Siglecs, meaning Siglecs act as immune checkpoints (244). So, any eosinophil-targeted IBD therapy, including Siglec-8 stimulation, will need careful monitoring. Perhaps intermittent dosing or localized delivery (e.g., enema formulations for UC) could mitigate risks. On the ROS side, therapies increasing ROS must avoid oxidative damage elsewhere; localized approaches (like a NOX1 agonist confined to the gut) would be needed, and ensuring antioxidant pathways are concurrently upregulated is important.
7. **Biomarkers**: If the model is correct, we might exploit it for biomarkers. For instance, measuring fecal or serum ECP/EDN is already a proxy for eosinophil activity in IBD. Perhaps assays for soluble Siglec-8 ligands or anti-Neu5Gc antibodies could indicate if the Siglec-8 pathway is engaged. A high anti-Neu5Gc titer might predict non-response to Siglec-8 ligand therapy (since those antibodies could block ligand). Conversely, low sialylation in mucins (detected by lectin binding tests on biopsies) might identify patients who lack the natural brake and thus are candidates for eosinophil-targeted treatment.

In conclusion, eosinophils occupy a peculiar niche in IBD—not as central as T cells or neutrophils, but as significant amplifiers of inflammation and facilitators of healing. The ROS-based model and Siglec-8 pathway provide a framework that reconciles these opposing roles. It emphasizes the importance of context: eosinophils can be friend or foe depending on the local signals and regulatory mechanisms. Therapies of the future might aim to tilt eosinophils toward the friend side—either by promoting their constructive functions or by selectively disarming them when they become destructive. Siglec-8 is a promising handle to achieve the latter, essentially telling eosinophils “enough, time to die” when they've served their purpose. As our understanding grows, interventions that once seemed counterintuitive (like using pro-oxidants or leveraging glycan signals) may become part of the armamentarium against IBD.

Ultimately, integrating allergology and gastroenterology insights—as this review has attempted—can generate novel hypotheses and therapies. By expanding our view of IBD pathogenesis to include eosinophils, ROS, and glycobiology, we open doors to innovative treatments that could complement existing immunosuppressants and biologics. The hope is that a more finely tuned modulation of the innate immune environment (such as tweaking eosinophil activity) will yield better long-term outcomes for patients with IBD, promoting not only remission of inflammation but also durable healing of the gut mucosa.

## Data Availability

The original contributions presented in the study are included in the article/Supplementary Material, further inquiries can be directed to the corresponding author.
